# Fitness preferential attachment as a driving mechanism in bitcoin transaction network

**DOI:** 10.1371/journal.pone.0219346

**Published:** 2019-08-23

**Authors:** Ayana Aspembitova, Ling Feng, Valentin Melnikov, Lock Yue Chew

**Affiliations:** 1 Division of Physics and Applied Physics, Nanyang Technological University, 21 Nanyang Link, Singapore, Singapore; 2 Institute of High Performance Computing, Agency for Science, Technology and Research, 1 Fusionopolis Way, Singapore, Singapore; 3 Department of Physics, National University of Singapore, 2 Science Drive 3, Singapore, Singapore; 4 Complexity Institute, Nanyang Technological University, 50 Nanyang Avenue, Singapore, Singapore; 5 Data Science and Artificial Intelligence Research Centre, Block N4 #02a-32, Nanyang Avenue, Nanyang Technological University, Singapore, Singapore; Universidad Rey Juan Carlos, SPAIN

## Abstract

Bitcoin is the earliest cryptocurrency and among the most successful ones to date. Recently, its dynamical evolution has attracted the attention of the research community due to its completeness and richness in historical records. In this paper, we focus on the detailed evolution of bitcoin trading with the aim of elucidating the mechanism that drives the formation of the bitcoin transaction network. Our empirical investigation reveals that although the temporal properties of the transaction network possesses scale-free degree distribution like many other networks, its formation mechanism is different from the commonly assumed models of degree preferential attachment or wealth preferential attachment. By defining the fitness value of each node as the ability of the node to attract new connections, we have instead uncovered that the observed scale-free degree distribution results from the intrinsic fitness of each node following a power-law distribution. Our finding thus suggests that the “good-get-richer” rather than the “rich-get-richer” paradigm operates within the bitcoin ecosystem. Based on these findings, we propose a model that captures the temporal generative process by means of a fitness preferential attachment and data-driven birth/death mechanism. Our proposed model is able to produce structural properties in good agreement with those obtained from the empirical bitcoin network.

## Introduction

Cryptocurrencies are new technological inventions that became extremely popular in just a few years, and by the end of 2018 their total market capitalization reached 205 billion USD. Unlike any other financial securities (currencies, bonds, stocks etc.) that are authorized and regulated by governments/companies, cryptocurrencies do not depend on any single authority and operate as peer-to-peer networks of users connected through the Internet. Bitcoin is the first and one of the most successful cryptocurrencies, proposed by an unknown individual or group using the name of Satoshi Nakamoto [1]. Several studies have contributed to a better understanding of the technology [2, 3], its evolution and dynamics [4–6], and associated financial risks [7, 8]. Current research has also uncovered scale-free behavior and the driving mechanism of degree preferential attachment [4]. Despite gaining a clear picture of the network structure from these studies, there is still a gap in understanding on which factors lead to the current structure of the bitcoin network, and why we observe certain properties and behaviour. Hence, in this paper, we attempt to uncover the driving mechanism behind the temporal evolution of the bitcoin network through a network analysis of its transactions.

Network analysis has proven to be a powerful tool to analyze and study problems in complex systems. Studies conducted along this direction have employed many theories and models with a static network approach to examine the properties of real-world systems. Despite the diversity of the systems investigated, which span the social [9–11], biological, and technological realms, the networks in these systems typically share a subset of similar statistical properties [12–14]. Nonetheless, many real-world complex systems are not static, and in the case of bitcoin we cannot simply treat it as a static network. Real-world systems evolve over time and change in size. New nodes and connections are constantly created, while at the same time the links and nodes that are redundant or unused are deactivated. A temporal network modeling approach is thus more suitable for many real-world systems because it incorporates dynamical information of the complex network topology and provides a fairer analysis of complex systems [15, 16]. Recently, temporal networks have attracted great attention and various modelling techniques were proposed [17–23]. In particular, temporal network approaches have been applied to problems in various fields, such as neuroscience [24] and epidemiology [25].

While we have investigated the temporal evolution of network properties of a real-world system, we have also examined its aggregated (static) network for a more thorough analysis of its macroscopic properties. Considering both the static and temporal properties of the system helps to get a more realistic understanding of the system. In consequence, we have employed both the modelling techniques of static and temporal networks to study the bitcoin system, and have discovered various interesting properties, such as power-law degree distributions, disassortative degree correlations, a linear growth of network, etc. Though the scale-free behaviour in the bitcoin system was discussed in previous research [5], [4], the driving mechanism behind it is still not understood. Our main research interest is to uncover how an innovative financial system evolves into a scale-free network. We empirically investigated various attachment models and conclude that fitness preferential attachment is the leading mechanism that drives the bitcoin system into scale-free behavior. We give the definition of fitness and discuss the behavior and evolution of fit nodes. Also, we propose a generative model to construct a temporal scale-free network that is close to the network of bitcoin transactions.

Overall, our paper is organized as follows. In Section I, we introduce the dataset and calculate the static and temporal properties of the bitcoin network. We then dedicate the next section of the paper to empirical investigation with the purpose of deducing the mechanisms in the network. In the subsequent section, we propose a generative algorithm for the bitcoin network and support our results with a synthetic network generated based on our proposed algorithm. We provide both a theoretical explanation of the network characteristics and a comparison between the model results and those derived from the empirical dataset. Finally, we conclude our findings in the last section.

## 1 Related work

There have been a few studies on bitcoin transaction data using tools from network science. One of the earliest studies on the bitcoin transaction graph is by Ron and Shamir [3] where the authors analysed the statistical properties of the graph as the number of links, distribution of addresses and the flows of the largest transactions. This work mainly focuses on understanding the structure of the bitcoin network and detecting behaviours as long chains, forks and binary tree-like distributions. Another exploratory study of the bitcoin network has been performed in [5] to show the basic statistics and dynamics of its network properties. The authors have reported the correlation between the user activity, transaction volume and the exchange rate of bitcoin. As for the network properties, the authors have found a scale-free degree distribution and a very large average path length. This work gave a preliminary understanding of the bitcoin graph in the initial stage of its development. Later research provided more insights into the system—in [4] the network characteristics together with their evolution over time were investigated. The authors have measured the wealth and degree distributions over time and found that the network is highly heterogeneous and assumed that nonlinear degree preferential attachment [26] might be the reason for these observed characteristics. However no direct investigation on their proposed mechanism has been made.

The power law degree distribution of the bitcoin network reported by the aforementioned researchers is a very interesting feature which shows that a decentralized innovative system still converges to a highly heterogeneous one. The question that remains open is what factors drive the bitcoin network into a scale-free one?

Scale-free behaviour is observed in diverse real-world systems and has been extensively studied. We call a network scale-free if its degree distribution follows a power-law, or in other words, when probability for node *i* to have a degree *k* exhibits a power-law distribution:

$$P\left(k_{i}\right)=ck_{i}^{-\alpha },$$ (1)

where *c* is some constant coefficient, and *k*_*i*_ is the degree of node *i*. Pioneering work of Barabasi and Albert proposed the concept of preferential attachment, where new nodes tend to connect to the nodes with higher degree rather than to nodes with less connections [27]. Modifications of this model have been able to capture many realistic features of complex networks—growth [28], rewiring [29], aging [30], initial attractiveness of the node [31], and node’s fitness [32]. The Dorogovtsev-Mendes-Samukhin model generalizes the Barabasi-Albert model by adding a parameter of the initial node attractiveness [31], while Krapivsky added a nonlinear term to define an attachment probability of the new node [26]. Bianconi and Barabasi have given a definition of fitness that captures the overall ability of a node to attract new links. Their model combines the impact of both degree and fitness to drive network connectivity [32]. Caldarelli replaced the preferential attachment rule based on degree, assuming that every node in a network has a fitness coefficient *x*, which is a random number taken from a given probability distribution. This model is somewhat close to the one proposed by Goh for static networks [33]. The fitness of a node represents its importance [34]. In [35], it was analytically proven that a power-law fitness distribution can be generated from any fitness distribution, with the simplest realization manifesting from the power law distributed fitness values. Dorogovtsev and Mendes showed that accelerated growth of links in a network (when the number of links grows faster than the number of nodes) may produce scale-free behavior [36]. The nonlinear growth has been observed in the WWW [37], Internet [38] and collaboration [39] networks.

Having a dataset with precise timestamps of link formation allowed us to directly investigate the underlying mechanism that leads to the scale-free behaviour. Based on our empirical findings, we constructed a generative algorithm that is able to reproduce the scale-free behaviour of the bitcoin network.

## 2 Empirical investigation of bitcoin transaction network

In this section, we describe our dataset, define the bitcoin transaction network, and analyze its properties.

### 2.1 Data description

Being the most popular cryptocurrency in the market, bitcoin has an extremely large size of transactions history (more than 100GB by the time we started this research). We used the initial four years of transactions to focus on the initial growth mechanisms that led the decentralised financial system to scale-free behaviour. The four year data set consists of more than 25 million transactions that were made among more than 5 million users.

As the first and one of the most successful cryptocurrencies, bitcoin has also attracted a lot of attention from researchers of different fields. In particular, Reid and Harrigan deanonymized users in the bitcoin system by constructing two networks: The transaction network and user network. They combined these two networks with additional external information and various techniques, such as context discovery and flow analysis. Details of this approach can be found in [2]. After identifying unique users in the bitcoin system, it became straightforward to construct a network with unique users as nodes and transactions as links between nodes. To get a set of data convenient for analysis, we used the code of Ivan Brugere [40]. This code employs the strategy of Reid and Harrigan to produce flat-file formats with all information retained, and covered the period from 01 January 2009 to 08 January 2013. Table 1 shows the structure of the data file, with each row of the file presenting information on the sender ID, receiver ID, time of transaction, and the amount of bitcoin transacted.

**Table 1:** An example of the dataset obtained using the Reid and Harrigan method [2]. The data contains information on the users’ ID (both sender and receiver), exact time, and amount of transaction.

Sender key	Receiver key	Date	amount
501194	2645	20101105221244	0.90
501194	502661	20101105221244	0.10
834628	834630	20100718123114	2.00
834628	536837	20100718123114	0.05
713610	5188	20110218223432	50.00

It is important to note that there are two phases (or periods) during the initial 4 years when bitcoin was used as a peer-to-peer cryptocurrency system. There is the first period (which lasts until the end of 2010), when it was mostly used as an experimental system. In the second period, it functioned closer to a real asset or currency. Prior research has found that this first and second period have different characteristics [4], [5]. In our investigation, we performed network analysis and simulations separately over each of these two periods.

### 2.2 Network properties of the bitcoin transaction data

From this dataset, we first constructed an aggregated static network where the nodes are unique users in the bitcoin system (even if a user has several wallets, they appear as one node in the network) and the links are transactions among them. Each new transaction was considered as a new link—if users transacted with each other multiple times, they will create multiple links between each other. We have derived two static networks from these transactions: one for the first period and the other for the second period. First, we have calculated the main properties of the static network representation, where Table 2 summarizes them as follows:

**Table 2:** Basic properties calculated for the static bitcoin network in the first and second period. An increased number of links and nodes in the second period shows that the bitcoin network had grown significantly by 2013. A larger clustering coefficient in the late period indicates a higher connectivity of the system users. The average degree also increased in the late period, showing that the system had become more popular as each node tended to create more connections in comparison to the first period (where most of the nodes transacted only a few times).

Property	First period (2009-2010)	Second period (2011-2012)
Number of nodes	193,012	4,689,069
Number of links	302,116	11,432,100
Global clustering coefficient	0.116	0.24
Average Degree	1.565	2.438
Assortativity coefficient	0	-0.1

**Fig 1:**
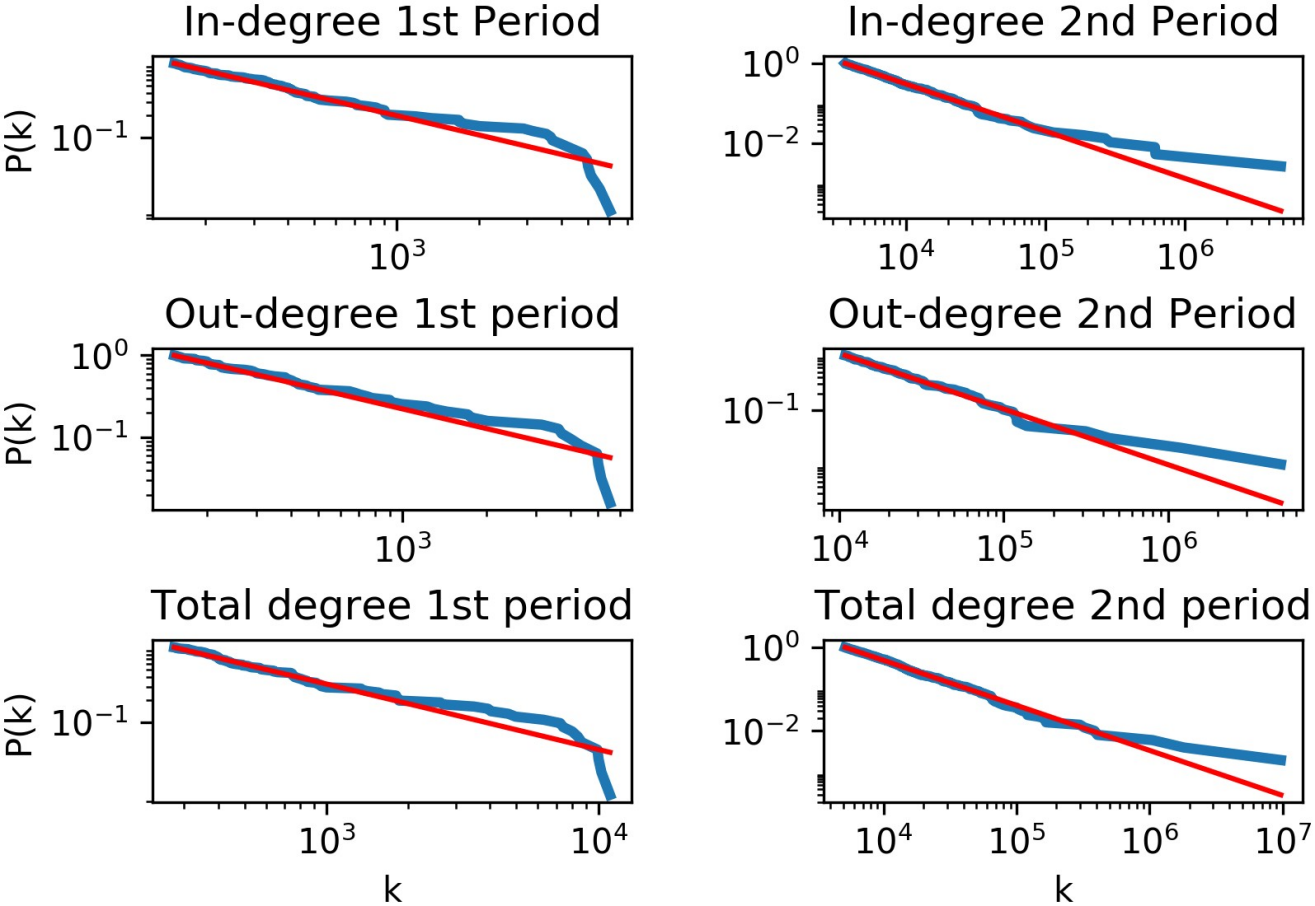
Degree distributions empirically calculated separately for the two periods and shown as blue lines in the form of complementary cumulative distribution functions (CCDFs). The red lines show a power law function with an exponent of -2.

Another important network property, degree distribution, was calculated and plotted for the two periods. Fig 1 shows that the in-degree, out-degree, and overall degree distributions resemble power-law functions.

Table 3 shows the calculated value of the power law exponent. Fittings and exponent calculations were made using the “power-law” python package [41], which implements the existing statistical methods of [42–44].

**Table 3:** Calculated values for *α* for the in, out, and overall degree distributions for the first (1P) and second (2P) periods.

	In (1P)	In (2P)	Out (1P)	Out (2P)	All (1P)	All (2P)
*α*	1.87	2.2	1.8	2	1.87	2.07

**Fig 2:**
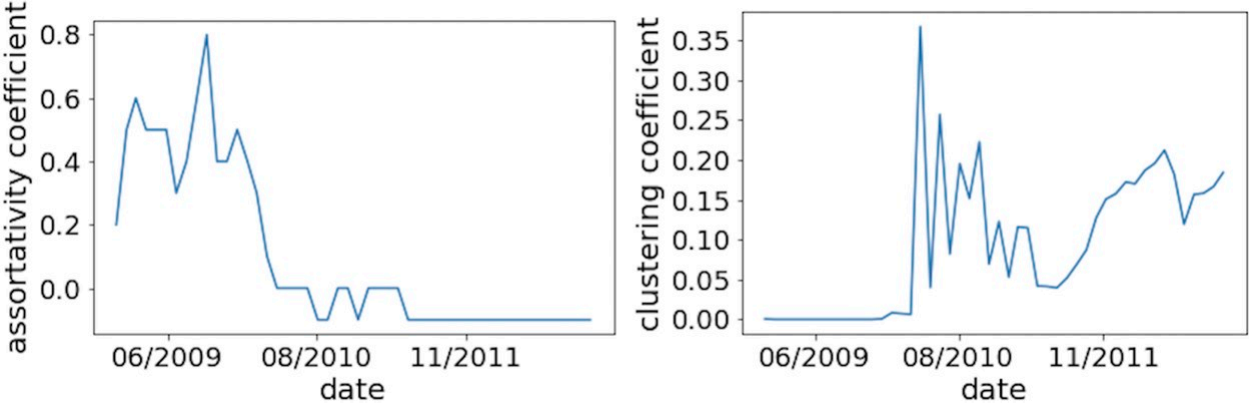
Assortativity and clustering coefficients calculated by month. The network is highly assortative in the first period and stabilizes to slightly disassortative behavior (-0.1) in the second period. In the beginning, the bitcoin network was not clustered. Then, wild fluctuations of the clustering coefficient are observed until the end of the first period. The second period shows a steady growth in the clustering coefficient.

**Fig 3:**
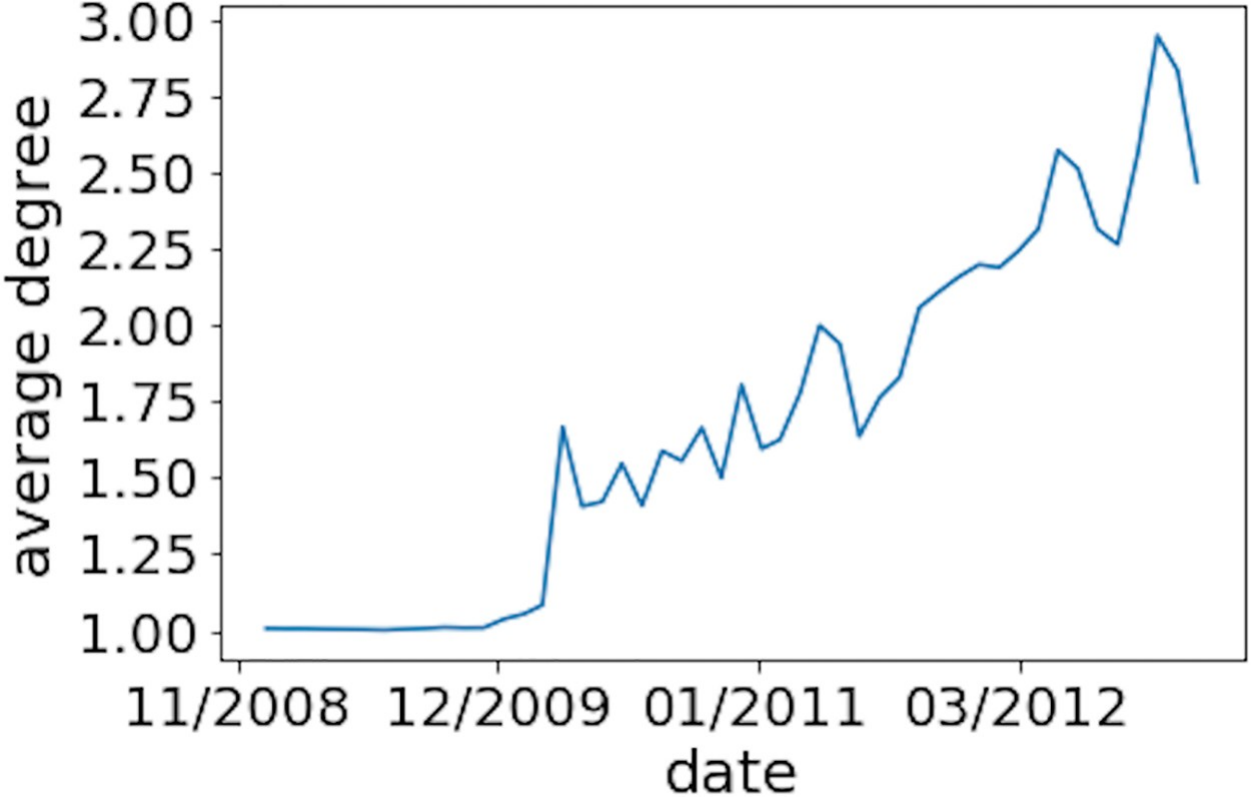
Evolution of the monthly network average degree. We observe that the average degree of the network grows steadily with time.

**Fig 4:**
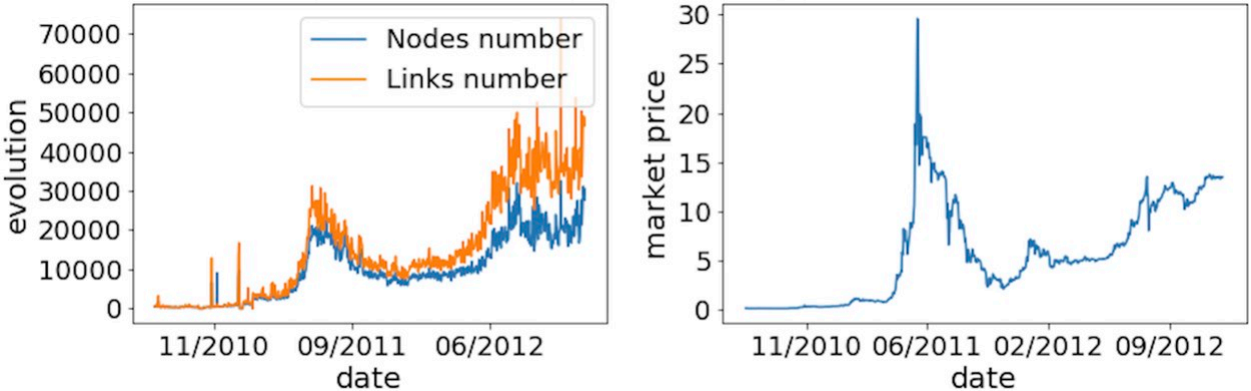
The daily evolution of the total number of nodes and links. The history of the bitcoin market price. Interestingly, the evolution of the total number of nodes and links shares some similarity with the evolution of the bitcoin market price with a peak in 2011. Correlation for nodes number/price and links number/price was calculated and is 91% and 92 % respectively.

To understand the network better, we expanded the analysis to include the time information of link creation. We represented the network as a sequence of smaller static networks, calculated the properties as for a static network, and observed the evolution of properties over time. We also calculated various properties using daily and monthly time windows. For properties such as assortativity, average degree, and clustering coefficient, a one-month time window allowed us to observe clear trends, while using a daily time window was too sparse to provide many meaningful insights. Fig 2 shows the evolution of the mixing patterns in the bitcoin network. As for the dynamics of the average degree (see Fig 3), we see its value grow steadily over time. For the evolution of nodes and links, the monthly network shows a general trend of steady growth, while the daily time window allows us to see that the network size is directly related to its price dynamics. (Fig 4 illustrates this relationship).

In the bitcoin network, the change from a high assortative to a low disassortative structure can be explained by the overall evolution of the network. During the early period the network is characterized by many self-looped nodes, big hubs, etc. After the bitcoin system had become developed, there could have been more people starting to use it for real transactions rather than for speculations, leading to a change towards disassortative behaviour. A possible explanation of this behaviour is that mining pools (represented as nodes with very high degree) were selling bitcoins to the others—higher degree nodes tend to connect to lower degree nodes. Also, people may have used bitcoins to transact with various companies and entities which explains the preference of low degree nodes to connect to those of high degree.

**Fig 5:**
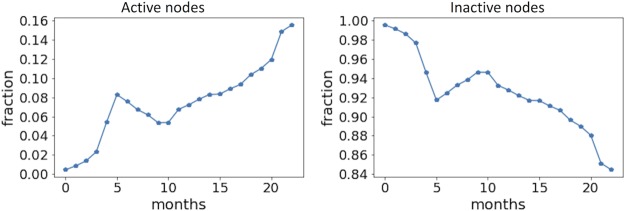
The monthly fraction of active and inactive nodes over the total number of nodes in the given month for the second (stable) period. It can be seen that most of the nodes (users) were not active every month.

We have also analysed the temporal dynamics of the number of active and inactive nodes in the bitcoin system. We considered a node as active in a given time period if it had created at least one new link, and inactive otherwise. Fig 5 shows that most of the existing nodes in the bitcoin network were inactive. However, as the system evolved, the fraction of inactive nodes decreased significantly.

**Fig 6:**
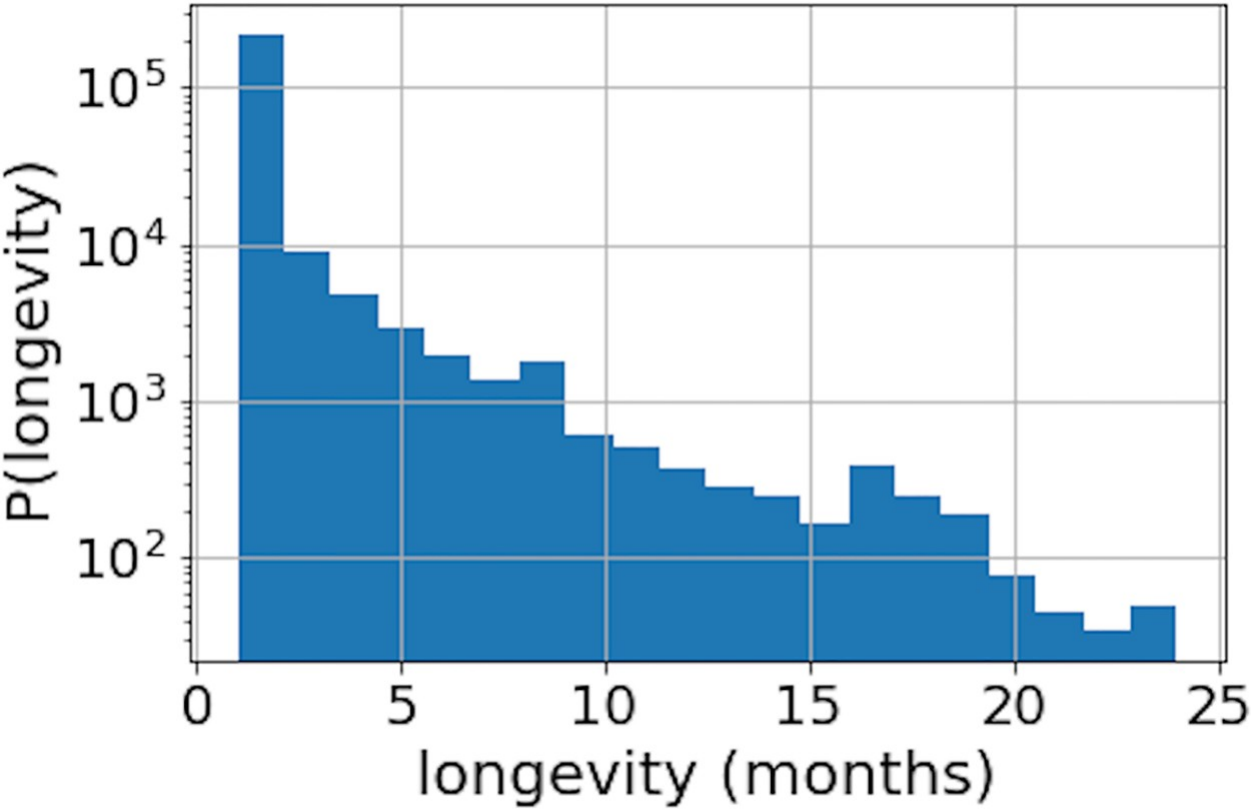
Longevity distribution for nodes in the second (stable) period. Most of the nodes stay in the network for a short period, while only a few nodes remained active for more than 20 months.

In addition to the analysis of active and inactive nodes, we also calculated the distribution of nodes’ longevity. We define the longevity of a node as the duration that it stayed active in the network (creating new transactions) before the last transaction. More specifically, in the bitcoin network we distinguish three states of nodes—active (transacted in a given time period), inactive (did not transact in a given period, but resurrected later) and dead nodes (disappeared from the network and never transacted again). Longevity for a node *i* was calculated as the total number of months when it was active (skipping the inactive months) before it becomes dead. Fig 6 illustrates that most of the nodes do not live long (stay active) in the bitcoin system.

Our network analysis above has revealed some interesting static and temporal properties of the bitcoin system—scale-free behavior, linear growth of the average degree, growing number of active nodes, short life-time of nodes, and high correlation between network size and market price. In the later section, we shall investigate the network’s generative mechanism.

## 3 Mechanism behind the bitcoin transaction network evolution

Network analysis revealed some interesting properties of the bitcoin system such as a power-law degree distribution and varying degree correlations. Scale-free behaviour is one of the common features of many real-world complex networks and it has been observed in various complex systems—social, communication, biological, etc. Many studies have proposed generative mechanisms for scale-free degree distributions in empirical networks. Here we investigate some of the major mechanisms using our dataset so as to uncover the real underlying mechanism that leads to a power-law degree distribution of the bitcoin transaction network.

**Fig 7:**
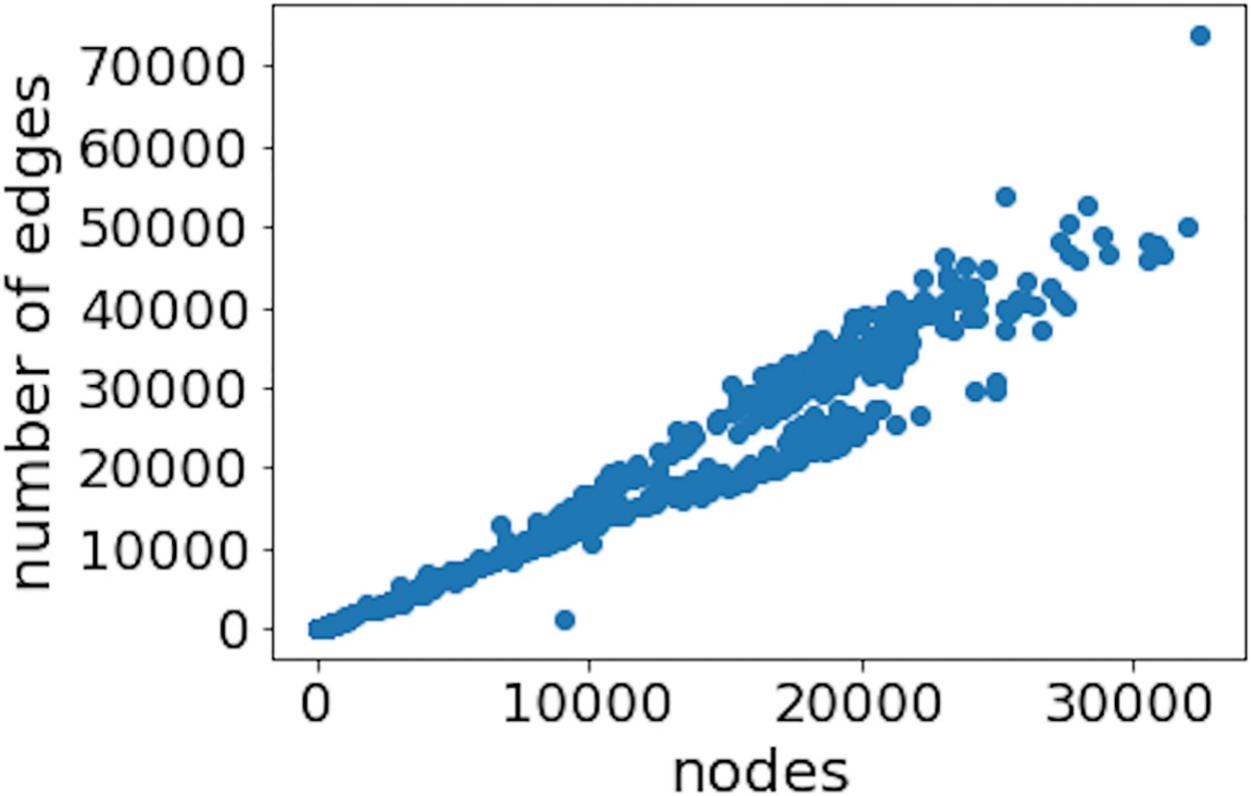
Relation between the number of nodes and links for daily data. It can be seen that the growth of links in the bitcoin network is approximately linear.

First, we investigate if the bitcoin network possesses an accelerated growth [36] which could be the cause of the observed power-law degree distribution. Fig 7 shows that the number of links grows roughly linearly in relation to the number of nodes. Therefore, in the bitcoin network, we do not observe the accelerated growth that occurs in the WWW, internet, or in collaboration networks.

Next we check if the scale-free behaviour results from the mechanism of preferential attachment. Nodes in different systems might have various preferences to connect to other nodes. Degree preferential attachment proposed by Barabasi and Albert [27] has assumed that new nodes tend to connect to existing nodes with higher degree rather than to those with less connections. Mathematically, the probability that a new node will connect to another node *i* is:

$$\begin{array}{c} p_{i}=\frac{k_{i}}{\sum_{l}k_{l}}. \end{array}$$ (2)

Later generalizations of this model [28], [29], [30], [31], [32] add more parameters but still assume that a node’s degree plays an important role in link creation.

For some complex systems, degree preferential attachment might be a reasonable explanation for the scale-free behaviour, while for other systems the information about the node’s degree is either not available or is not an important factor for other nodes to connect to. In the bitcoin network, it is also reasonable to assume that richer nodes could attract more connections. Therefore, we might observe the mechanism of wealth preferential attachment, where the probability that new node will connect to node *i* is:

$$\begin{array}{c} p_{i}=\frac{w_{i}}{\sum_{l}w_{l}}, \end{array}$$ (3)

where *w*_*i*_ is the amount of bitcoins in the wallet of node *i*.

**Fig 8:**
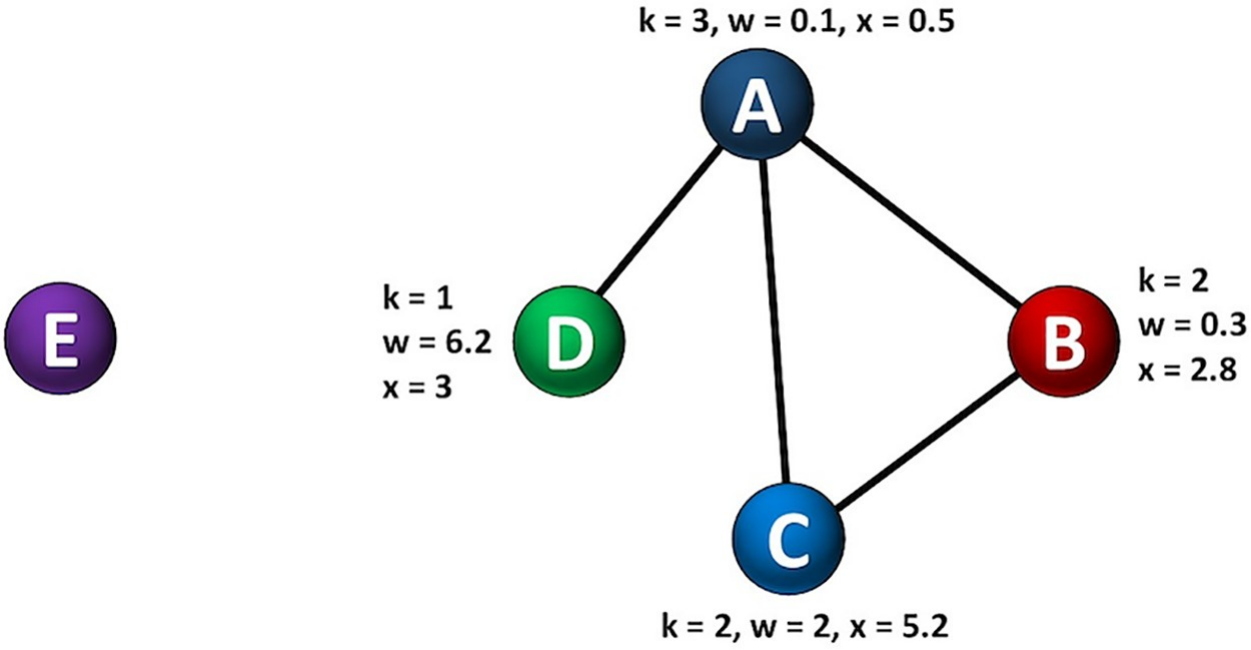
Illustration of a toy network. Each existing node in the network (nodes A, B, C, D) has properties such as degree *k* (how many transactions were made), wealth *w* (amount of bitcoins in wallet), and fitness *x* (the potential of node *i* to create new connections, where different types of users in the system have different intrinsic fitness. A new node E might chose to connect to any existing node depending on its preference—based on degree, wealth or fitness.

Another possible factor to create new connections might be the type of user in the bitcoin network. For example, exchanges and businesses would have more chance to become hubs, while common users’ accounts would have less potential to have many transactions. For the intrinsic potential of a node to attract new links in a network, we define the characteristic of the *fitness* of a node. The fitness coefficient depends on the type of user in the bitcoin network and therefore, does not change significantly over time. Fig 8 demonstrates a toy network, where each node has three properties—degree, wealth and fitness.

**Fig 9:**
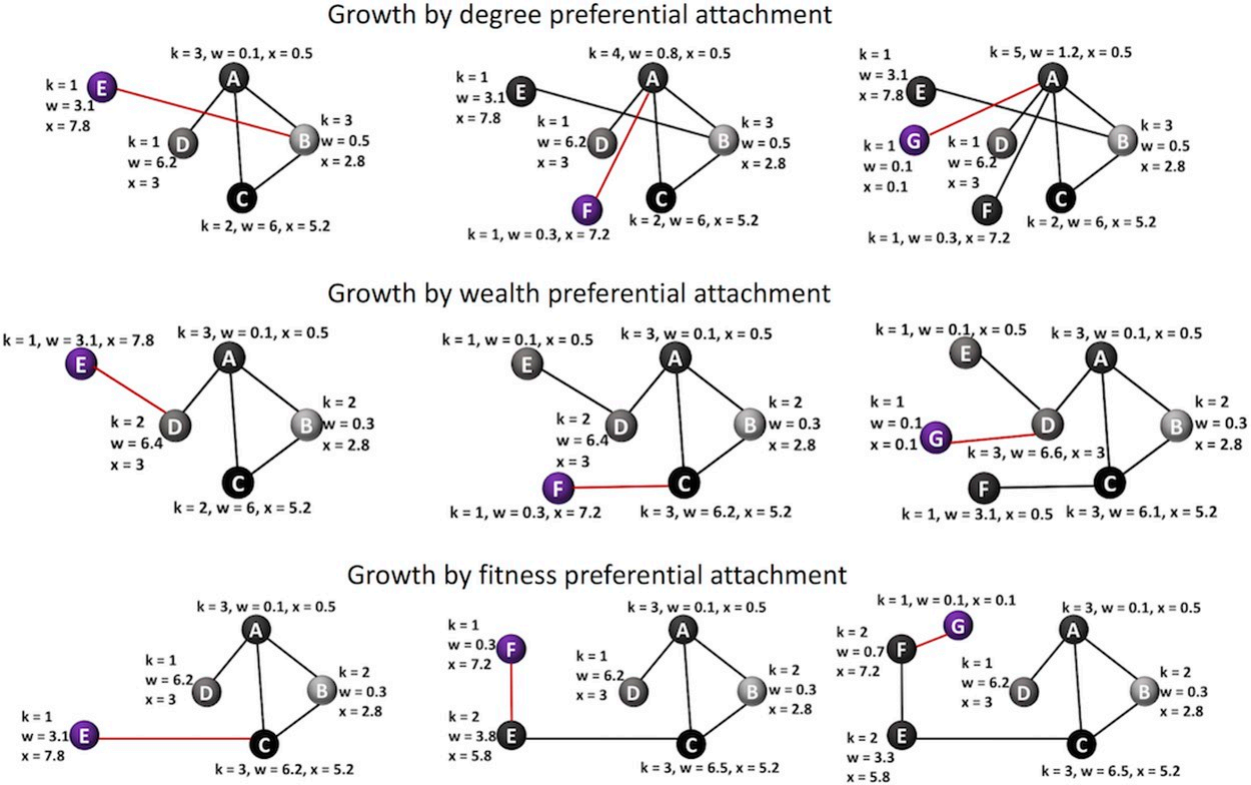
Illustration of the growth of toy network based on various attachment mechanisms.

Fig 9 shows how a toy network evolves based on different attachment mechanisms. For simplicity, one new node is added at each time step with other links remain static and unchanged.

### 3.1 Degree attachment mechanism

To see if there is degree preferential attachment in our network, we empirically check how a node’s degree affects its ability to create connections. Having precise time steps of interactions in our dataset allowed us to derive the statistics of attachment preferences. We checked transaction by transaction if there were new nodes (users) that entered the system. For every new node we determined the degree of the node to which it was attached. As we surmise that the attachment mechanism of the new nodes to the existing ones might differ from the mechanism of how old nodes connect with each other, we have examined the attachment mechanism separately for new and old nodes for both periods. For a fair comparison, we have normalized the overall distribution of degree attachment statistics.

**Fig 10:**
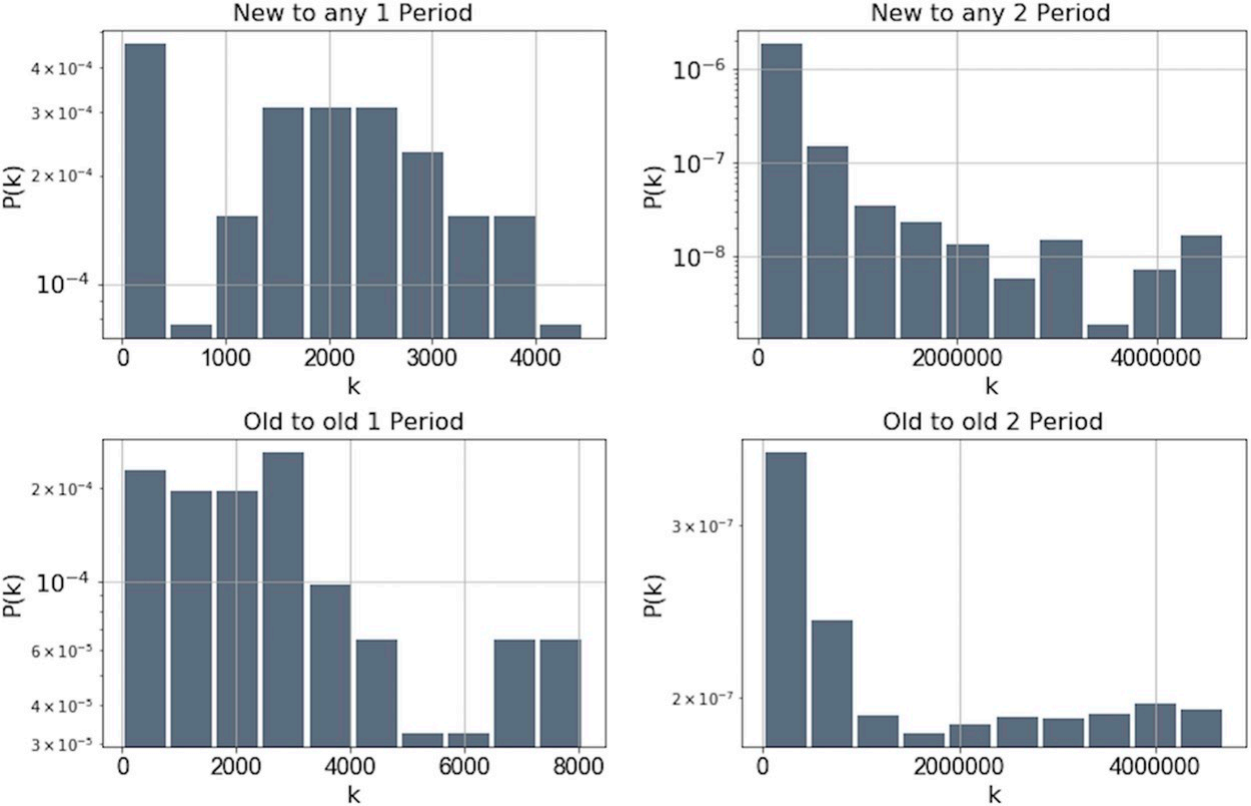
Normalized degree attachment probabilities to new and old (existing) nodes in both periods. We can see that for both new and old nodes, there is no clear preference to attach to the nodes with higher degree.

Fig 10 shows the results of our analysis, which indicates that there is no preference for nodes to attach to high-degree nodes. Moreover, we found that in the second period, the preference to connect to nodes with smaller degree is more pronounced than to higher degrees. Based on this finding, we conclude that in the bitcoin network, degree preferential attachment is not the generative mechanism that drives the bitcoin network towards a scale-free degree distribution.

### 3.2 Wealth attachment mechanism

Since the bitcoin network is directed and links are weighted, it is possible to track the amount of bitcoins in each wallet. For an additional analysis, we checked if attachment preference depends on the node’s wealth.

**Fig 11:**
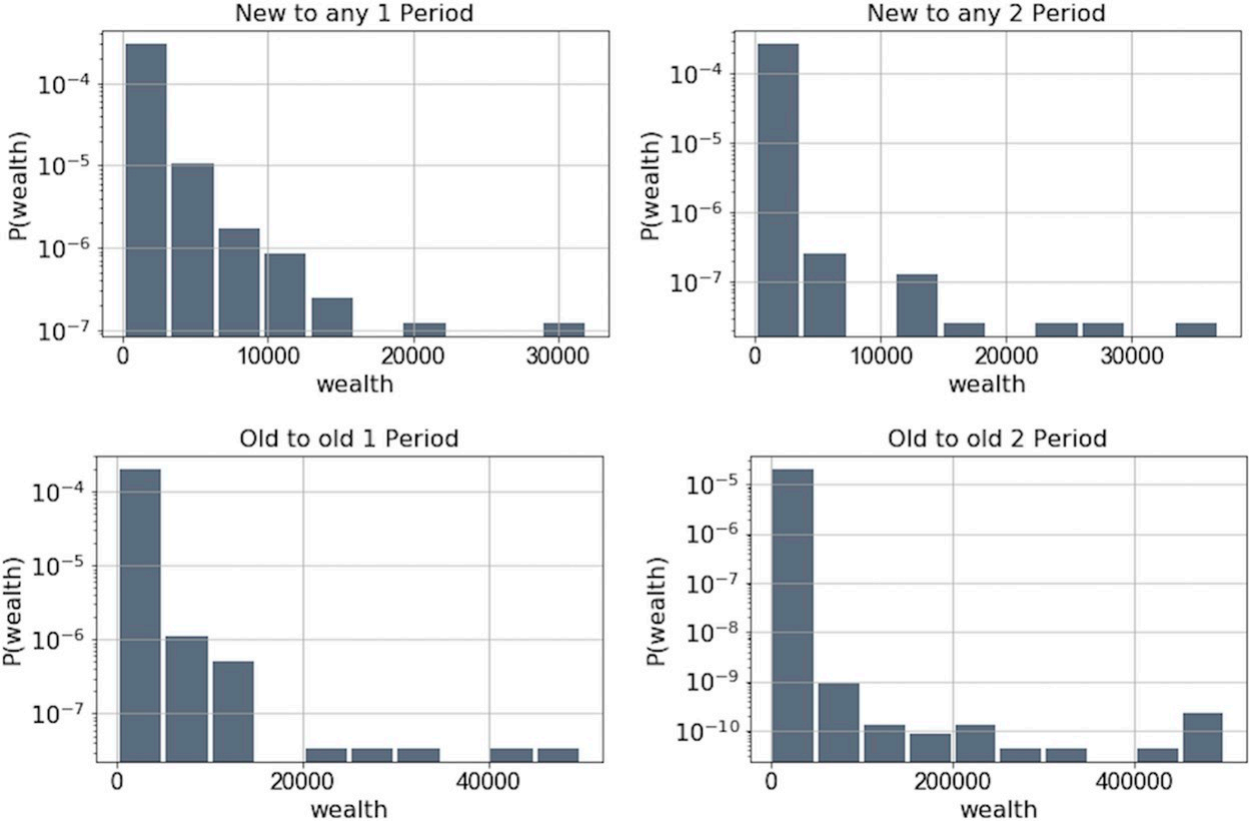
Normalized wealth attachment probabilities for attachment to new and old (existing) nodes in both periods. We can see that for both new and old nodes, there is no clear preference to attach to the richer nodes.

The analysis was normalized by the total amount of bitcoins transacted in each period. From Fig 11 we can see the clear preference in both periods that nodes do not tend to transact with other nodes that have higher amounts of bitcoins in their wallets.

### 3.3 Fitness distribution of network nodes

As it was shown, there is no clear dependency between the degree (or wealth) of a node and its ability to create new links in the bitcoin network. We empirically calculate the fitness of a node as its probability to attract new connections at each time step:

$$\begin{array}{c} x_{i,n}=\frac{k_{i,t=n}-k_{i,t=n-1}}{\sum_{j=1}^{m}(k_{j,t=n}-k_{j,t=n-1})}\;, \end{array}$$ (4)

where *k*_*i*,*t*_ is the degree of node *i* at time *t*, *n* = 1, 2, …, 23 is the month of the given period, and *m* is the number of nodes in the network.

We found that there are two distinct patterns in node fitness: first, some fit nodes do not live long in the network—they create relatively more transactions in a short period and then disappear. We would attribute this behaviour pattern to the speculative users of the system. Another behaviour type observed in nodes with high fitness is high longevity and constant creation of new links. This type of node might be exchanges, businesses that accept bitcoin payments, etc.

**Fig 12:**
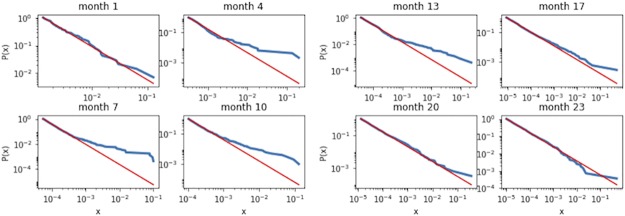
The fitness distributions computed for all nodes month by month in the second (stable) period of bitcoin transactions and shown in CCDF (blue line). The Red line shows the power-law function with an exponent of -2. Considering the limitation of space, we show the fitness distributions for every 3 to 4 months. It can be seen that the distribution has a power-law shape with some deviation from month 4 until month 13.

We have calculated fitness values for all the nodes for each month over the second period, as shown in Fig 12. Due to limited space, we have only shown the results for each month at an interval of three to four months. Fig 12 reveals that the distribution of the nodes’ fitness has essentially the shape of a power-law except for the period from month 4 to month 13. The observed deviation from power-law for these months arises from a hike in the transaction activities of bitcoin with a corresponding rise in the number of nodes and links, and price fluctuations (see Fig 4). Once this transient spurt of interest is over, the fitness distribution of the nodes was found to settle back into a power-law distribution. Fig 12 displays the complementary cumulative distribution function of the nodes’ fitness (blue line) plotted with a power-law fit (red line). The exponent of the power-law has a value of -2. Note that nodes with zero fitness were removed from the analysis because of their inactivity during the given months.

**Fig 13:**
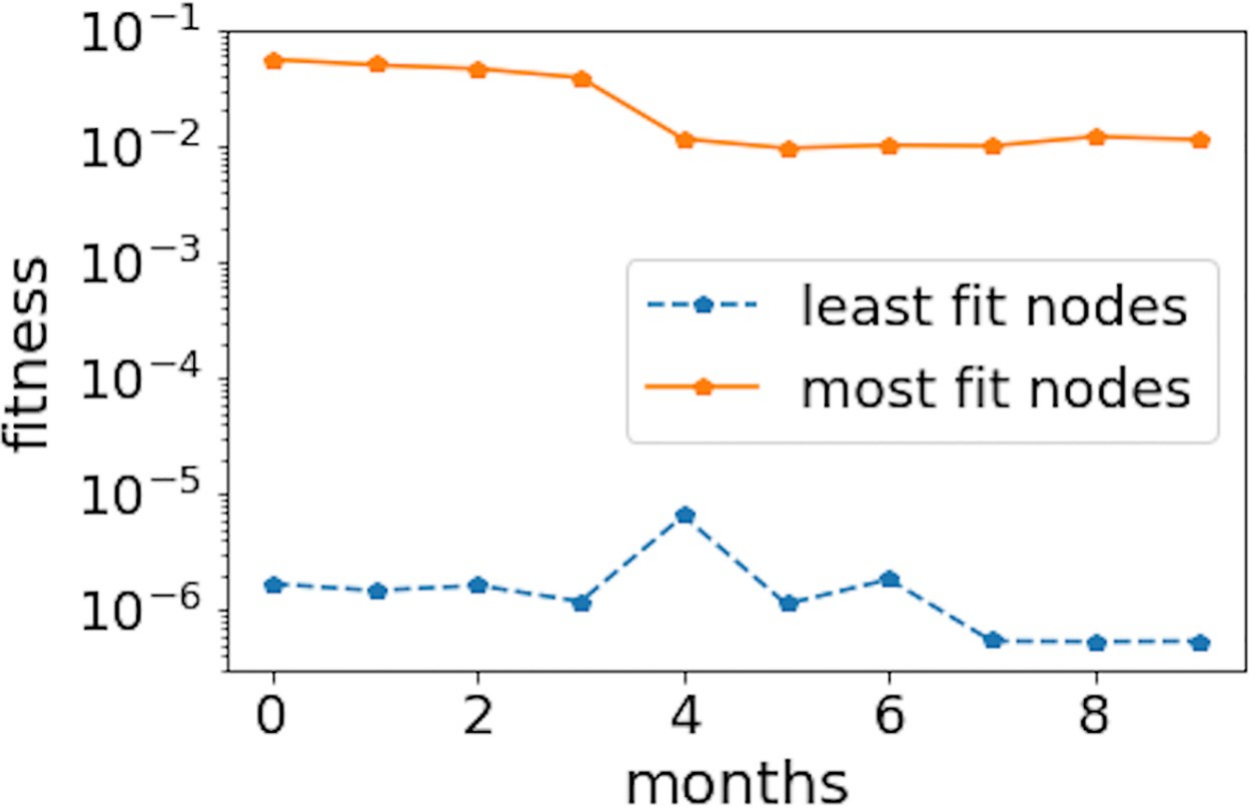
Evolution of the fitness coefficient for the most-fit and the least-fit nodes. The average fitness coefficient was calculated for the most- and least-fit nodes in the beginning of the period and we tracked the change until the end of the period. It can be seen that the fitness coefficient does not change significantly over time.

To understand if the fitness coefficient of a node remains approximately constant during its lifetime (or if the coefficient changes significantly instead), we have looked into the evolution of the most-fit and the least-fit nodes. For the most-fit nodes, we have chosen the top ten nodes in our study because these nodes have significantly higher fitness than the rest in the network. In our investigation on the evolution of the least-fit nodes, we have chosen a sample of 3, 500 nodes since these nodes all have the smallest fitness coefficient from the beginning of our observation. Fig 13 shows the evolution of the average fitness coefficient for the most-fit and least-fit nodes, where we observe a level of fitness that remains relatively constant over time. This finding supports our definition of fitness (intrinsic potential to create new connections that depends on the type of user) in the bitcoin network due to the nature of the nodes—exchanges remain to be exchanges while common users do not become traders or businesses over time.

The age of a node is an indicator of the temporal nature of a network. In a static network, nodes are immortal. On the other hand, the age of a node is finite in a temporal network. As a result, we are interested in how the degree and the fitness coefficient of a node are related to its age. One might expect that a long-lived node would have a higher degree, or a larger fitness coefficient. However, we found a low correlation between the age of a node and both its degree and fitness throughout the period of study (see Table 4). The correlation between fitness and degree is also lower than expected for a network with links created by fitness preferential attachment. These lowered correlation can be explained by the fact that while high fitness nodes create many links in a short span of time, these nodes become inactive quickly and do not have a high degree by the end of the period. This dynamical and topological behavior of the nodes is not surprising in a fast evolving financial network of transactions.

**Table 4:** Correlation between the fitness, degree, and age for all the nodes in the network by the end of the second (stable) period. We found the correlation between degree and age, and between fitness and age, to be small.

	Fitness	Degree	Age
Fitness	-	0.47	0.03
Degree	0.47	-	0.02
Age	0.03	0.02	-

In summary, our empirical analysis on the bitcoin transaction network has revealed a power-law-like distribution of nodes’ intrinsic fitness which does not change significantly over time. On the other hand, we observed that nodes in the network typically have a short life span.

## 4 Generative algorithm for the bitcoin transaction network

In the previous section, we have investigated the driving mechanism of the bitcoin network that could potentially lead to the observed scale-free behaviour (with a power-law degree distribution), but we found that the degree or wealth of a node is not the cause of link creation in the bitcoin network. On the other hand, we have uncovered that nodes possess intrinsic fitness which is power-law distributed, and we expect this to bring about a power-law degree distribution. In the study of Caldarelli [34, 35], it was shown that scale-free behavior might arise from any fitness distribution with the simplest realization being fitness coefficients following a power-law. The approach of Caldarelli has inspired our creation of a generative algorithm for the bitcoin transaction network, with the generated network yielding both the static and temporal properties of the real-world system.

### 4.1 Algorithmic model for bitcoin network generation

The algorithmic steps of the model are as follows:

1. Create a large set of seed nodes with links to initiate the synthetic network. For every node *i*, assign a fitness coefficient *x*_*i*_ randomly from a power-law distribution. The value of *x*_*i*_ reflects the node’s ability to create new connections.
2. Simulate the entrance of new nodes to the network. This is the birth process.
3. Randomly create links between new-to-new nodes. On the other hand, links between new-to-old nodes and old-to-old (existing) nodes are created with old nodes being assigned a number of links proportional to their level of fitness.
4. Assign fitness coefficients for the new nodes so that the fitness distribution remains power-law distributed.
5. Define the fraction of nodes that will be inactive for the next time step. Select these inactive nodes randomly according to the given fraction and assign a fitness coefficient of zero to them. This is the death process.
6. Repeat the algorithm from step 2.

Note that steps 2 and 5 emulate a fundamental property of temporal networks—the networks do not only grow with time, they can also shrink. Nodes are not constantly active, they might be temporarily inactive or even leave the network. For nodes that have become inactive but return to an active state later, we call them resurrected nodes. If the inactive nodes never turn active again, we consider the nodes to be dead.

A feature of our generative algorithm is its incorporation of our empirical findings, where links are created according to fitness preferential attachment, and the fitness coefficients of nodes are power-law distributed (which do not change significantly over time). Also, we have implemented the birth and death processes by capturing the evolution of nodes in the bitcoin system.

### 4.2 Synthetic network simulations results

**Fig 14:**
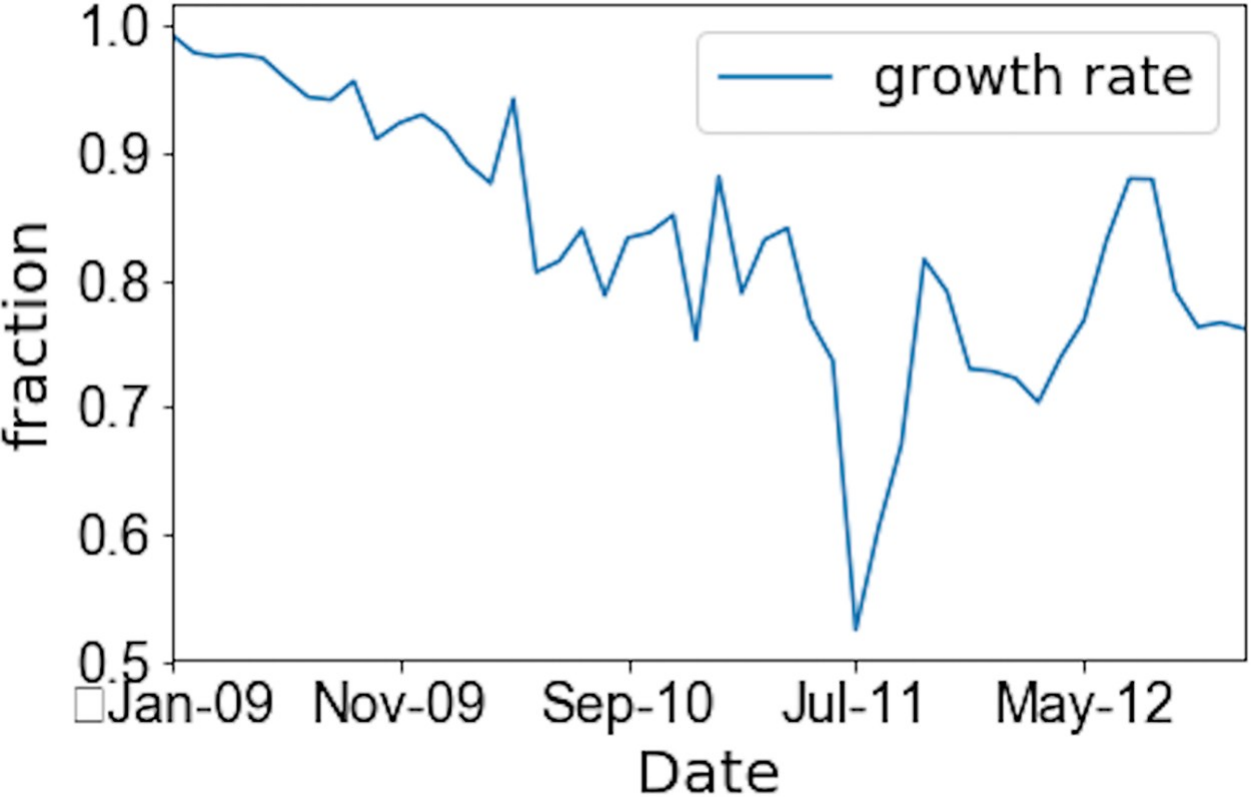
Evolution of the network growth rate *R*. This figure shows the fraction of new nodes entering the system at a given month over the total number of active nodes in that month for both periods.

We have created synthetic temporal networks based on the proposed algorithm. During the generation process, we used the initial network from the empirical data at the start of each given period (first and second) as seeds to start our simulations. The next step of network generation is to include new nodes. The number of nodes to be added at each time step is defined according to the empirical growth rate as follows:

$$\begin{array}{c} R=\frac{n_{new}}{n_{total}}\;, \end{array}$$ (5)

where *n*_*new*_ is the number of new nodes entering the system at a particular month, while *n*_*total*_ is the total number of nodes in the same month. Fig 14 shows the evolution of this network growth rate *R* over four years.

As described in 3.1, we calculated the statistics of nodes to which new ones were attached. From there we observed that in the real bitcoin network approximately 2/3 of new nodes are attached to the other new ones (degree of zero at the moment of attachment), while 1/3 of new nodes are attached to the existing ones (degree greater than zero).

We followed this same *rule* in our network generation process. Furthermore, the number of new links we created at each time step has been set to be consistent with the average degree of the empirical dataset—thus for each new node created, we add 1.57 links for the first period, and 2.44 links for the second period.

**Fig 15:**
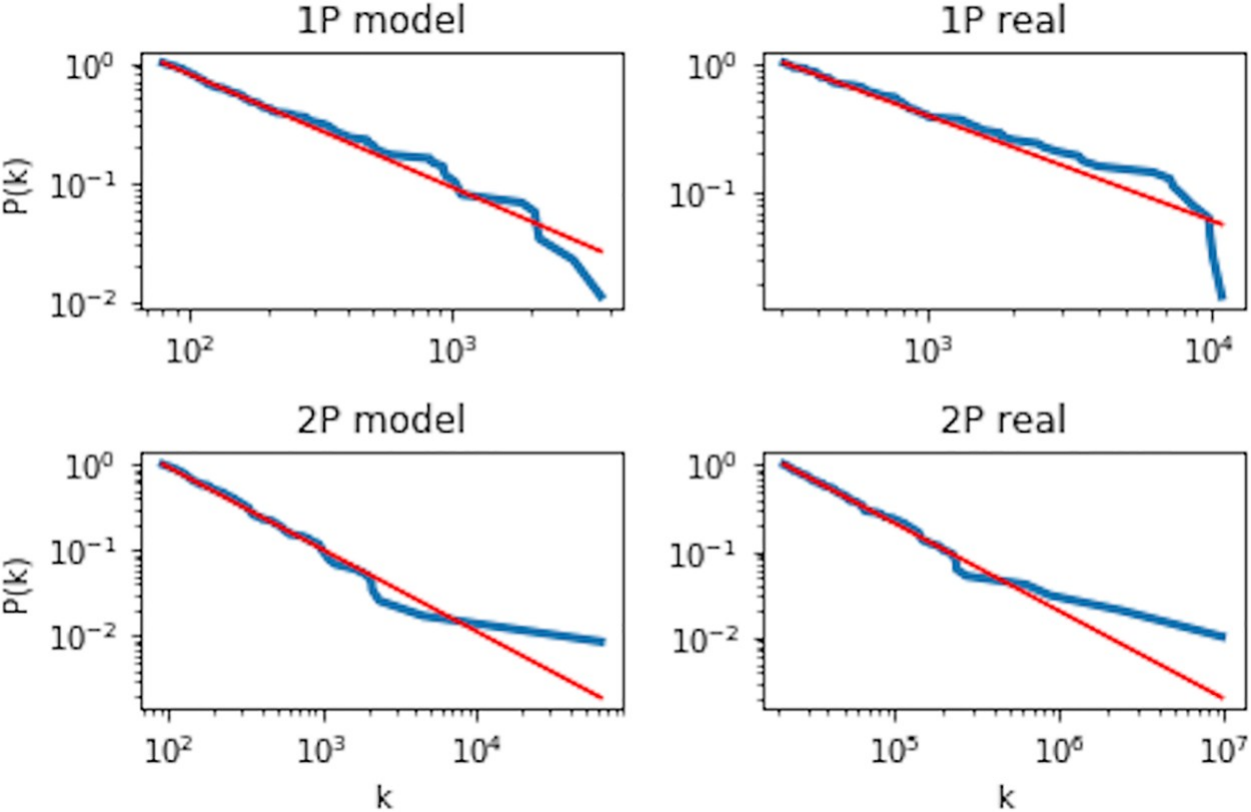
A comparison of the complimentary cumulative degree distribution between the empirical and synthetic network for both periods. All distributions are observed to display power-law behavior. The red line shows the power law function with an exponent of -2.

We then assigned fitness coefficients randomly to the new nodes of the synthetic network according to an empirical fitness distribution. After which, we randomly set a fraction *f* of the network nodes to the inactive state according to the empirical findings in Fig 5. We repeated the algorithm, and after running for 24 time steps, the synthetic network was found to evolve towards a structure with a power-law degree distribution. We calculated the nodes’ degrees of our synthetic network by aggregating the in- and out-degrees, thereby treating the network as undirected. A comparison of the degree distribution of our synthetic network with the empirical bitcoin network shows good correspondence in scale-free behaviour (see Fig 15). In particular, it is worth noting that the exponent of the degree distribution around -2 is close to that of the fitness distribution. This is expected since the fitness coefficients directly determine new link creation for the node *i*. The similar exponent value with those observed in the empirical network thus validates the assumptions of our model.

Subsequently, we generated an ensemble of 10 synthetic temporal networks for the evaluation of their average network structural properties. Table 5 compares these network properties between the synthetic and the bitcoin transaction network. The scale-free behaviour is well captured by the model, while the clustering coefficient differs between the real and model networks. A higher clustering coefficient in the real network might arise from the social aspects—recommendations, following the choice of neighbours, etc. Our model simulates connections without capturing these social aspects and therefore, we observe the model network to be less clustered. For the assortativity coefficient, our simulation was able to capture the decreasing trend from first to second period, although the value slightly differs.

**Table 5:** A comparison of basic network properties between the synthetic and empirical bitcoin network. We observe that while our model was able to reproduce the scale-free behaviour and diassortativity of the empirical network (for the 2 periods), the clustering coefficients still differ. We relate it to the social aspects of the bitcoin system which were not implemented in the current model.

Property	Model	Empirical
Number of nodes (1st Period)	193,012	193,012
Number of nodes (2nd Period)	4,689,069	4,689,069
Number of links (1st Period)	302,985	302,116
Number of links (2nd Period)	11,441,315	11,432,100
Clustering coefficient (1st Period)	0.08 ±0.02	0.116
Clustering coefficient (2nd Period)	0.12 ±0.03	0.24
Average Degree (1st Period)	1.57	1.57
Average Degree (2nd Period)	2.44	2.44
Assortativity coefficient (1st Period)	-0.05 ±0.01	0
Assortativity coefficient (2nd Period)	-0.08 ±0.01	- 0.1
Power law exponent (1st Period)	1.94.	1.98
Power law exponent (2nd Period)	2.16	2

**Fig 16:**
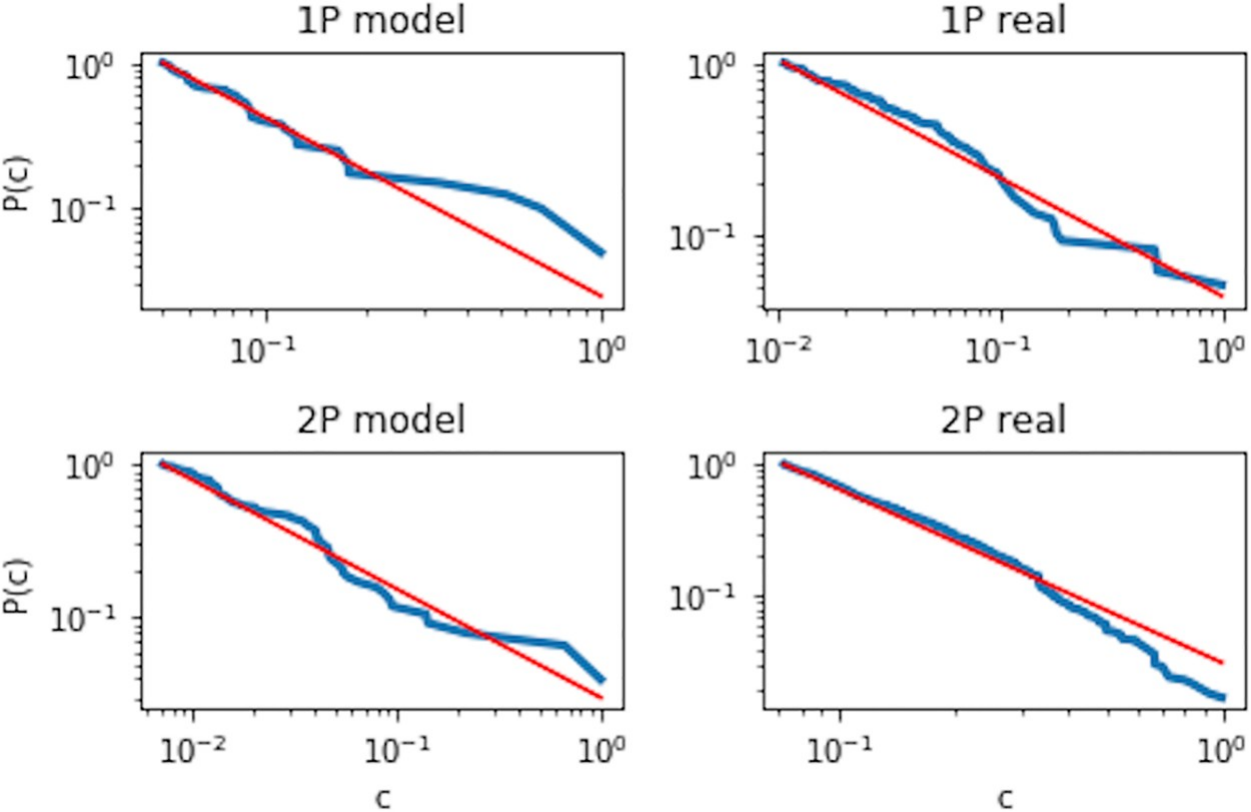
A comparison on the clustering coefficient distribution between the empirical and synthetic network for both periods. All distributions are observed to display power-law behavior. The red line shows the power law function with an exponent of -2.

To further validate our model, we have performed analysis on the other topological properties of the synthetic network to see how close they are to the empirical network. For this, Fig 16 illustrates our results on the complementary cumulative distribution of the clustering coefficient *c*(*k*) versus node degrees. Note that *c*(*k*) was calculated by averaging over the clustering coefficient of all nodes in the network that possess the same degree *k*. We found that the clustering coefficient is power-law distributed for both periods, and for both the empirical and the synthetic networks. In the first period, we observed that *c*(*k*) ∝ *k*^−1.68^ for the synthetic network, and *c*(*k*) ∝ *k*^−2.2^ for the empirical network. On the other hand, during the second period, we found that *c*(*k*) ∝ *k*^−1.71^ and *c*(*k*) ∝ *k*^−2.32^ for the synthetic and the empirical network respectively.

## 5 Methods

### 5.1 Bitcoin transaction network

Bitcoin operates as peer-to-peer network of users connected through the internet. Approval of payments is guaranteed by making the transaction history transparent—every user can observe and download the previous transaction records and provides proof/validation that a certain transaction took place. The anonymity of users is ensured by providing the possibility to create as many wallets (addresses) with different IDs as needed. It is recommended to create a new wallet for every transaction. The blockchain with all the transaction history can be downloaded after installing the open-source bitcoin client. Alternatively, websites such as blockchain.com provide transaction history in a more convenient format. However, due to anonymity in the bitcoin system, analyzing raw data does not give us a fair understanding of the network structure. A few methods were developed to deanonymize bitcoin clients—by discovering IP addresses [45] and by clustering bitcoin addresses [2]. The first method is effective but extremely resource intensive, which makes it infeasible on a large scale. The clustering method exploits multi-input transactions and assumes that input addresses in one transaction belong to the same user. This method requires less computational power and makes the deanonymization process feasible.

Ivan Brugere implemented the method proposed by Reid and Harrigan [2] in his code [40] and was able to obtain a deanonymized transaction data set which is publicly available [46].

### 5.2 Data

We obtain the bitcoin transaction dataset from the website [46] by Brugere. This data includes more than 25 million links (transactions) and more than 5 million nodes (users).

## 6 Conclusions

In this work, our detailed network analysis of the bitcoin transaction network has provided insights into its network structural properties and the underlying evolution mechanisms. We found that the in-degree, out-degree, and the overall degree distributions during the first and second periods display scale-free behavior. Also, we have uncovered the dynamics of different structural properties which yielded a change in the set of network characteristics over the two periods. In particular, the assortativity and clustering coefficients were observed to stabilize before the second period, while deviation from the power-law of the fitness distribution happened a few months before the sudden increase in transaction activities and market price.

Our empirical study on the attachment mechanism of the bitcoin transaction network showed that there is neither degree preferential attachment of nodes nor wealth preferential attachment in the system, although certain nodes are intrinsically better at attracting links due to properties not observed in their local network properties. We surmise that the power-law behavior observed in the degree distributions results from the nodes’ intrinsic fitness, i.e., its intrinsic ability to create new connections. This could be related to the types of users—exchanges are more attractive to connections than active traders, who are then more attractive than a common adopter of bitcoin. Our empirical analysis on the distribution of node’s fitness of the bitcoin network indicates a power-law distribution that is relatively stable over time. By generating synthetic temporal networks based on this fitness distribution and further incorporating the birth/death of nodes, we have demonstrated that the scale-free behavior of the degree distribution, as well as other topological properties, are consequences of the mechanism of fitness preferential attachment.
